# Attributable fraction and number of cancer cases attributed to occupational carcinogens in Iran in 2019

**DOI:** 10.1186/s12889-026-28144-z

**Published:** 2026-06-25

**Authors:** Ahmad Naghibzadeh-Tahami, Yahya Khosravi, Ahmad-Reza Zamani, Ali-Akbar Haghdoost

**Affiliations:** 1 Neuroscience Research Center, Institute of Neuropharmacology, Kerman University of Medical Sciences, Kerman, Iran; 2 Department of Occupational Health and Safety Engineering, School of Health, Alborz University of Medical Sciences, Karaj, Iran; 3 Physiology Research Center, Institute of Neuropharmacology, Kerman University of Medical Sciences, Kerman, Iran; 4 HIV/STI Surveillance Research Center, WHO Collaborating Center for HIV Surveillance, Institute for Futures Studies in Health, Kerman University of Medical Sciences, Kerman, Iran

**Keywords:** Neoplasms, Attributable fraction, Occupational carcinogens, Iran

## Abstract

**Background:**

Although occupational exposure to carcinogenic agents accounts for only a relatively small fraction of cancers, the estimated burden in Iran remains considerable due to widespread exposure in major industries and the scarcity of national data. This study aimed to estimate the proportion and number of cases of bladder, leukemia, lung, laryngeal, and liver cancers attributable to four major occupational carcinogens—silica, benzene, asbestos, and lead—in Iran in 2019.

**Methods:**

An attributable fraction (AF)-based approach was used to estimate the burden of occupational cancers in Iran. AFs were calculated using odds ratios (ORs) associated with specific occupational exposures. Although AFs are traditionally calculated using relative risks (RRs), ORs were used because they are more commonly reported in occupational studies and can approximate RRs when outcomes are rare. For each exposure, ORs were selected from epidemiological sources, including industry- or population-based studies, meta-analyses, and reviews. The number of workers exposed to occupational carcinogens in 2019 was obtained from the national occupational exposure surveillance system. AFs were then applied to national cancer incidence data to estimate the attributable numbers (ANs) of cancers related to occupational exposures. Monte Carlo simulation was used to estimate uncertainty intervals (UIs) for both AFs and ANs.

**Results:**

In 2019, 60 (95% UI: 46–77) cases of lung, bladder, liver, laryngeal, and leukemia cancers were attributable to historical lead exposure (AF = 34%). The total number of cancer cases attributable to historical silica exposure was estimated at 623 (95% UI: 365–881) (AF = 36%). Furthermore, 602 (95% UI: 334–887) cases of bladder, lung, liver, leukemia, and laryngeal cancers were attributable to occupational benzene exposure (AF = 23%). For asbestos, the AF was 42%, but the estimated attributable number was very small because of the limited number of exposed workers (AN = 0.41, 95% UI: 0.264–0.538).

**Conclusion:**

These findings suggest that occupational exposure to carcinogenic agents may contribute substantially to the burden of selected cancers in Iran. Although the estimates are exploratory and based on scenario-based modeling assumptions, they highlight the importance of preventive workplace interventions and improved occupational exposure surveillance systems.

**Supplementary Information:**

The online version contains supplementary material available at https://doi.org/10.1186/s12889-026-28144-z.

## Introduction

In occupational settings, a wide range of chemical agents are present, as they are either intentionally used in industrial processes or generated as by-products [1]. These agents can enter the human body through inhalation, dermal absorption, ingestion, or, less commonly, injection, with inhalation and dermal absorption representing the predominant occupational exposure routes [2, 3]. Such exposures are associated with a wide range of adverse health outcomes, including respiratory diseases, allergies, developmental disorders, musculoskeletal and cardiovascular diseases, as well as cancer and adverse perinatal outcomes [4].

According to the International Agency for Research on Cancer (IARC) Monographs, nearly 200 agents are classified as carcinogenic to humans (Group 1) or probably carcinogenic to humans (Group 2 A), a substantial proportion of which are encountered in occupational settings [5–8]. Global estimates indicate that approximately 19% of all cancers are attributable to environmental and occupational factors, accounting for nearly 1.3 million deaths annually, with a substantial proportion related to workplace exposures [7, 9–12]. Major occupational carcinogens include asbestos, crystalline silica, lead, and benzene, which are causally associated with cancers of the lung, bladder, larynx, liver, and the hematopoietic system [13, 14].

Evidence from low- and middle-income countries, including Iran, indicates increasing occupational exposure to carcinogenic agents such as BTEX compounds, crystalline silica, and heavy metals, alongside ongoing industrial expansion, suggesting a potentially increasing future burden of occupational cancer [15–17] .For instance, although the use of asbestos in final products was banned in 2000 and the import of white asbestos was prohibited in 2012, some industries, such as automobile manufacturing, continued to rely on asbestos-containing materials for several subsequent years [18].

In Iran, national cancer registry data indicate substantial incidence of lung, bladder, leukemia, liver, and laryngeal cancers annually [19], while occupational exposure surveillance systems report ongoing exposure to key carcinogens among workers, including crystalline silica, benzene, lead, and asbestos [20].

Although the proportion of exposed workers is relatively small, previous studies suggest that these exposures may contribute disproportionately to the cancer burden and may increase further with industrial development. Two previous Iranian studies based on secondary data have estimated occupational cancer risks, reporting non-negligible attributable fractions for lung and leukemia cancers associated with occupational exposures [21, 22].

AFs are widely used to quantify the contribution of a specific risk factor to disease occurrence and to inform priority setting and targeted prevention strategies [8]. However, the validity of AFs estimates strongly depends on the availability of reliable data on exposure prevalence and effect sizes, which are often limited in low- and middle-income countries. Estimating AFs for occupational carcinogens requires reliable effect size measures—typically relative risks (RRs) or odds ratios (ORs)—for each exposure–cancer pair, as well as accurate estimates of the number of exposed workers over the relevant latency period. In the present study, ORs were used as valid approximations of RRs, given the relatively low incidence of the cancers considered.

AFs estimates are not directly comparable across countries due to differences in selected carcinogenic exposures, cancer outcomes, exposure prevalence, and methodological approaches. For example, previous studies have varied in the number and classification of included IARC agents, ranging from 25 to 44 exposures across different settings [7, 23, 24].

Similarly, estimates of occupational exposure prevalence, such as for crystalline silica, vary substantially between countries, reflecting differences in industrial structure and measurement methods [24]. These differences highlight the importance of country-specific burden estimates based on locally relevant data.

According to the Statistical Yearbook of Iran, approximately 1,703,800 individuals were employed in the industrial sector, the majority of whom were men [25]. However, no comprehensive quantitative estimation of occupational cancer burden attributable to major carcinogens has been previously conducted using a unified analytical framework in Iran. In settings where data are incomplete, scenario-based approaches can provide an initial quantitative approximation of potential burden rather than definitive estimates.

Therefore, this study aimed to estimate the attributable fraction and number of cancer cases associated with four major occupational carcinogens classified by IARC (crystalline silica, benzene, asbestos, and lead) for selected cancer sites (lung, bladder, leukemia, liver, and larynx) in Iran, using a scenario-based modeling approach to inform future surveillance and prevention strategies.

## Method

### Model

Using a stochastic model, we estimated the number of cancer cases attributable to four common occupational carcinogenic exposures, including silica, asbestos, benzene, and lead.

#### Input parameters to the model

We applied an attributable fraction (AF) approach informed by Levin’s method [26] to estimate the burden associated with each occupational carcinogen and cancer outcome (Eq. 1).

1 $$\mathbf{Equation \, 1:AF=\left(I_{exp}-I_{unexp}\right)/ I_{exp}}$$

Where Iexp is equal to the incidence in the exposed group and Iunexp is equal to the incidence in the non-exposed group. Other input parameters to the model included the number of workers exposed to carcinogens, ORs, and incidence in exposed workers and non-exposed people. The estimated AF was multiplied by the number of exposed workers to estimate the attributable number (AN) of cancer cases in the exposed population.

#### Data sources for incidence in exposed and unexposed groups

Baseline incidence rates for bladder, lung, laryngeal, and liver cancers and leukemia were obtained from the Iranian National Cancer Registry (2019). Incidence in the exposed group was estimated by applying the pooled ORs to these baseline national rates. The number of exposed workers for each carcinogen was extracted from the Ministry of Health’s national occupational exposure surveillance system. These datasets provided the required incidence estimates for both exposed and unexposed groups used in the AF calculations.

#### Assumptions of models

In this study, the modeling framework was designed to generate scenario-based estimates rather than direct empirical burden estimates. The input parameters were derived from heterogeneous data sources and combined under a set of structural assumptions (e.g., linear relationships, fixed population structure, and absence of interaction effects). Therefore, the model outputs should be interpreted as exploratory estimates reflecting the behavior of the model under the specified assumptions, rather than as precise epidemiological measures.

The uncertainty intervals (UIs) were obtained by propagating the variability of selected input parameters (i.e., ORs and the number of exposed workers) through Monte Carlo simulation. These intervals represent model-based uncertainty and do not capture the full spectrum of epidemiological uncertainty, such as residual confounding, exposure misclassification, or structural model assumptions.

The meta-regression analysis was performed solely to assess the consistency of effect sizes across cancer types for each exposure and to justify the application of pooled ORs within the modeling framework. It was not intended to generate new pooled epidemiological risk estimate.

### Data collection

#### A multi-step evidence identification approach was used

First, expert consultations were conducted to identify occupational carcinogens relevant to the Iranian context. These consultations were used only to refine the list of exposures and were not used in the analytical calculations.

Second, a scoping review was performed to map the range of occupational carcinogens and their associated cancer sites and to identify the most relevant exposure–cancer pairs for the Iranian setting. This step was purely exploratory and was used to guide the subsequent quantitative evidence synthesis.

Third, a systematic review was conducted to obtain quantitative effect estimates. Two independent reviewers performed a structured literature search, applied predefined eligibility criteria, and extracted adjusted ORs with 95% CIs. These estimates were used as inputs for the attributable fraction model. To evaluate whether a single pooled effect estimate could be applied across cancer types for each exposure, a random-effects meta-regression was performed.

#### Expert opinion

To address parameters for which reliable national data were not available, we conducted expert opinion interviews with individuals involved in the national occupational exposure surveillance program. Using purposive sampling, we recruited 25 experts who participated in semi-structured interviews to identify the most relevant occupational carcinogens in Iran and to provide estimates for missing exposure parameters. Expert opinions were used only in the preliminary phase to identify the most relevant occupational carcinogens and to define plausible ranges for missing exposure parameters; no expert-derived estimates were used in the final analytical calculations. All interviews were conducted in accordance with the Helsinki Declaration, approved by the Kerman University of Medical Sciences ethics committee, and performed after obtaining informed consent.

#### Scoping review

A scoping review was conducted to determine the types of main carcinogenic occupational exposures and the most common cancers attributed to these exposures [14]. This step allowed us to refine the list of exposures before extracting effect estimates from systematic reviews, ensuring that the selected ORs were applied to the most pertinent carcinogen-cancer pairs. Thus, the scoping review was used as a preparatory step to guide the data extraction process.

#### Systematic review and study selection

Two independent reviewers conducted a structured literature search in international and national databases, including PubMed, Scopus, Web of Science, Magiran, SID, IranDoc, and IranMedex. The search covered publications from January 2000 to September 2021.

The following search strings were used:

- “occupational exposure” AND “cancer risk” AND (“silica” OR “benzene” OR “asbestos” OR “lead”).
- “occupational carcinogens” AND (“lung cancer” OR “bladder cancer” OR “laryngeal cancer” OR “leukemia” OR “liver cancer”).
- “IARC Group 1” OR “IARC Group 2 A” AND “risk estimate” AND “occupational exposure”.

Inclusion criteria were [1] human studies; [2] cohort or case–control designs; [3] reporting adjusted OR with 95% CI; and [4] exposure specifically linked to one of the four carcinogens of interest.

Exclusion criteria included [1] animal or experimental studies; [2] ecological studies; [3] articles without adequate exposure assessment; and [4] reviews without extractable risk estimates.

Reference lists of included articles and relevant IARC Monographs were also screened.

#### Effect size synthesis and meta-regression

The ORs used for calculating the AFs were identified and extracted from eligible studies. When multiple estimates were available, ORs adjusted for major confounders were preferred. Because separate risk estimates for each exposure–cancer pair were not consistently available, a random-effects meta-regression was performed to assess whether the effect of each exposure was comparable across cancer types. Cancer type was entered as a covariate; the pooled estimates showed generally comparable effect sizes across cancer types, with no substantial change in the pooled ORs or between-study variance (τ²). The pooled ORs derived exclusively for this meta-regression assessment are presented in Supplementary Table S1.

#### Number of exposed workers

Based on the Ministry of Health’s occupational exposure surveillance system, 196,854 workers exposed to chemical carcinogens in 2019 were included in the study. In the national occupational exposure surveillance system, only a single value is reported for the number of exposed workers. Based on consultations with occupational health experts at the Ministry of Health, this reported figure represents the maximum estimate, while the minimum estimate is approximately 25% lower. We therefore defined an exposure range using these expert-informed minimum and maximum values.

#### Statistical analysis

##### Calculating the AF

Data from a variety of sources were combined and summarized. We used Eq. 1 to calculate the AF for each occupational carcinogen.

##### Estimating AN

ANs of cancers for the most recent year available at the time of the study for a specific cancer site were estimated by multiplying the AF by the total number of exposed workers that was obtained from the surveillance system of occupational exposures of the Ministry of Health of Iran.

Based on available data, industrial workers were aged 18–60 years, with an average age at employment of approximately 30 years. Assuming that their life expectancy is slightly lower than that of the general population (77 years), we considered it to be 70 years. Thus, workers were assumed to be exposed for an average of 40 years, during which attributable cancer cases could occur.

##### Confidence interval for the AF & AN

A 95% uncertainty interval was calculated for each AF, based only on the confidence interval (CI) of the OR estimates and variability in the number of the exposed workers used, and using Monte Carlo simulation methods where more than one OR estimate was involved [27]. A total of 1,000 replicates were generated [28] from a log-normal distribution, with the standard deviation (SD) derived from the point estimate and standard error (SE) for each OR and the number of exposed workers. These replicates were then used to estimate AF and AN. The 97.5 and 2.5 percentiles from this sample of AFs gave the 95% UI [29]. All simulations were performed using Microsoft Excel (Office 16, Microsoft Corp., Redmond, WA, USA).

### Sensitivity analysis

To evaluate the robustness of the estimated attributable cancer cases, we performed a sensitivity analysis based on different assumptions about under-reporting in the national cancer surveillance system. In the first scenario, assuming 100% reporting sensitivity, we considered that all cancer cases were correctly captured by the registry. In the second scenario, assuming 50% reporting sensitivity, we considered that approximately half of the true cancer cases were missed by the surveillance system, a level consistent with expert opinion regarding under-ascertainment in some regions of Iran. A substantial increase in the estimated number of cases under the 50% scenario would indicate that the results are highly sensitive to under-reporting. Conversely, if the change in estimated cases remained proportional, it would reflect a linear relationship, indicating that any degree of under-reporting would lead to a corresponding decrease in the estimated number of attributable cases [30, 31].

### Ethical approval

The ethics committee of Kerman University of Medical Sciences (KUMS) approved the study, with the ID number IR.KMU.REC.1399.407.

## Results

The findings of the scoping review have been published elsewhere [14]. Briefly, based on a scoping review, expert opinions, and the literature, the most common occupational cancers were bladder, lung, leukemia, laryngeal, and liver cancers. The most frequent occupational chemical carcinogens, according to the IARC monographs (established or probable), include asbestos, silica, benzene, chromium, cadmium, lead, diesel engine exhaust, and formaldehyde. In Iran, exposure to silica, asbestos, lead, and benzene is particularly common in various industries.

Table 1 presents the studies included in the systematic review and meta-analysis, examining the association between four major occupational exposures (lead, silica, asbestos, and benzene) and five common occupational cancers (lung, bladder, laryngeal, liver, and leukemia).

Therefore, for occupational exposures, three studies [32–34] assessed lead, five studies [35–39] assessed silica, six studies [35–37, 39–41] assessed asbestos, and three studies [42–44] assessed benzene.

**Table 1:** Studies related to occupational carcinogens included in the analysis and Odds Ratio (OR) for cancers associated with these carcinogens

Cancer	Agent	OR (95% CI)	Reference for OR
Lung	Lead	1.2(0.9–1.7)	Liao (2016)
Lung	Lead	1.4 (0.6–3.2)	Roussea (2007)
Liver	Lead	1.34 (0.71–2.25)	Heinemann (2000)
Lung	Silica dust, crystalline	1.35 (1.03–1.77)	Guida (2013)
	Asbestos	1.46 (1.17–1.83)	
Lung	Silica dust, crystalline	1.31(1.0–1.71)	Matteis (2012)
	Asbestos	1.78 (1.46–2.18)	
Larynx	Silica dust, crystalline	1.96 (0.8–4.4)	ELCI (2009)
	Asbestos	1.0 (0.6–1.5)	
Lung	Silica dust, crystalline	1.37 (1.14–1.56)	Cassidy (2007)
Bladder	Silica dust, crystalline	1.27 (1.0 −1.61)	Lidija (2020)
	Asbestos	1.58(1.2–2.08)	
Lung	Asbestos	1.28 (1.02–1.61)	Villeneuve (2012)
Larynx	Asbestos	1.6 (1.0–2.5)	Berrino (2003)
Lung	Benzene	1.35 (0.99–1.84)	Warden (2018)
Bladder	Benzene	1.16 (1.04–1.31)	Hadkhale (2017)
Leukemia	Benzene	1.24 (0.97–1.59)	Sorahan (2005)

Table 2 shows the estimated number and proportion of bladder, lung, leukemia, laryngeal, and liver cancer cases that could potentially be prevented if occupational exposures to silica, lead, benzene, and asbestos were eliminated in Iran. 

**Table 2:** Estimated attributable fractions (AFs) for all investigated cancer outcomes combined, and attributable numbers (ANs) by exposure and cancer site under the study assumptions

Carcinogen	Leukemia, AN (95% UI)	Lung, AN (95% UI)	Bladder, AN (95% UI)	Liver, AN (95% UI)	Larynx, AN (95% UI)	Total, AN (95% UI)	AF % (UI)
Silica	127 (79–177)	174 (114–248)	204 (128–299)	59 (36–85)	59 (37–84)	623 (365–881)	36 (26–44)
Benzene	122 (26–265)	168 (24–348)	200 (44–409)	56 (10–116)	56 (11–118)	602 (334–887)	23 (5–39)
Lead	14 (8–20)	16 (9–25)	19 (11–29)	6 (3–8)	5 (3–8)	60 (46–77)	34 (24–44)
Asbestos	0.079 (0.051–0.104)	0.122 (0.079–0.16)	0.152 (0.098–0.199)	0.033 (0.021–0.044)	0.023 (0.015–0.030)	0.41 (0.264–0.538)	42 (18–60)

AFs represent the combined attributable fraction across the five cancer outcomes included in this study (lung, bladder, leukemia, liver, and laryngeal cancers) for each occupational exposure. ANs are presented separately for each cancer type and as totals

 In 2019, an estimated 623 (95% UI: 365–881) cancer cases were attributable to historical silica exposure. These included 127 (95% UI: 79–177) cases of leukemia, 174 (95% UI: 114–248) cases of lung cancer, 204 (95% UI: 128–299) cases of bladder cancer, 59 (95% UI: 36–85) cases of liver cancer, and 59 (95% UI: 37–84) cases of laryngeal cancer.

We found that 36% (95% UI: 26–44) of lung, bladder, liver, laryngeal, and leukemia cancer cases among workers in Iranian industries could potentially be prevented by eliminating exposure to silica.

Based on the results shown in Table 2, a total of 602 (95% UI: 334–887) cancer cases were attributable to historical benzene exposure.

We found that bladder cancer, with 200 (95% UI: 44–409) cases, had the highest number of cases attributable to occupational benzene exposure. This was followed by lung cancer with 168 (95% UI: 24–348) cases, leukemia with 122 (95% UI: 26–265) cases, liver cancer with 56 (95% UI: 10–116) cases, and laryngeal cancer with 56 (95% UI: 11–118) cases.

Furthermore, approximately 23% (95% UI: 5–39) of lung, bladder, liver, laryngeal, and leukemia cancer cases among workers could be prevented by eliminating exposure to benzene.

The results showed that a total of 60 (95% UI: 46–77) cancer cases were attributable to historical lead exposure. This included 14 (95% UI: 8–20) cases of leukemia, 16 (95% UI: 9–25) cases of lung cancer, 19 (95% UI: 11–29) cases of bladder cancer, 6 (95% UI: 3–8) cases of liver cancer, and 5 (95% UI: 3–8) cases of laryngeal cancer. Furthermore, the results showed that 34.0% (95% UI: 24–44) of the total cases of lung, bladder, liver, laryngeal, and leukemia cancers among workers in Iranian industries may be attributable to lead exposure. If occupational lead exposure were eliminated, approximately 34% of cases of these cancers among workers in Iranian industries could potentially be prevented (Table 2).

According to the national occupational exposure surveillance system, only 57 workers were reported to be exposed to asbestos in Iranian industries in 2019. Although the AFs for asbestos across the five cancers (lung, bladder, liver, larynx, and leukemia) was estimated at 42% (95% UI: 18–60), the corresponding ANs for asbestos were extremely small due to the very limited number of exposed workers. The total number of asbestos-attributable cases (AN) was approximately 0.41 (95% UI: 0.264–0.538), reflecting the very limited number of exposed workers. Nevertheless, these very small expected values have been reported in Table 2 for completeness and to prevent any potential misinterpretation.

### Sensitivity analysis 

**Fig. 1:**
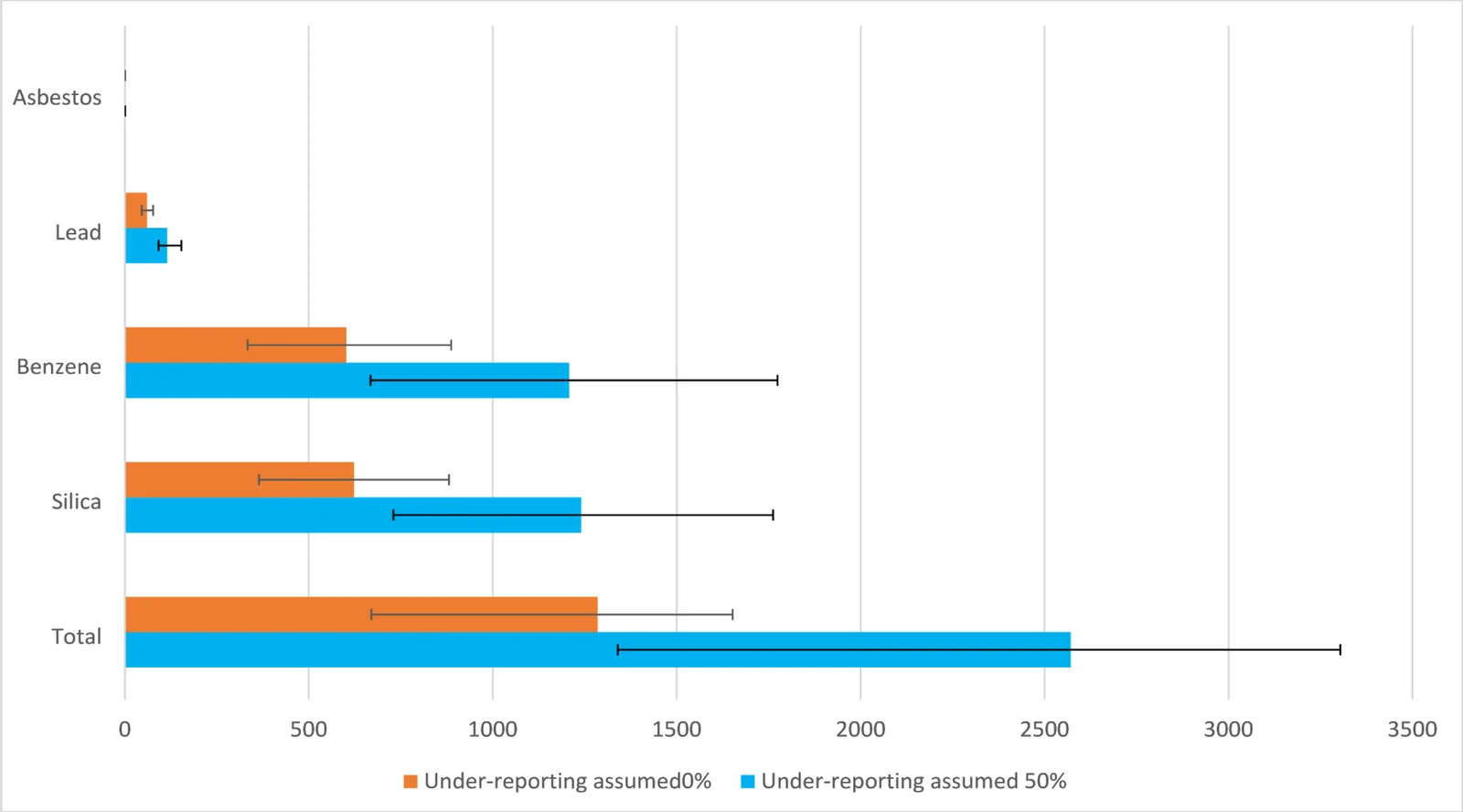
Total estimated additional cases of lung, bladder, liver, larynx and leukemia cancers attributed to all exposures during 40 years in two scenarios, reporting occupational exposure

Figure 1 presents the estimated total number of cancer cases attributable to four occupational exposures—silica, lead, asbestos, and benzene—over a 40-year period. Assuming 100% reporting sensitivity in the occupational exposure surveillance system, the total number of attributable cancer cases was estimated at 1,285.

In contrast, in the sensitivity analysis assuming a reporting sensitivity of 50%, the estimated number of cancer cases attributable to these four exposures increased to 2,571. Additional details are provided in Fig. 1.

## Discussion

This study is among the few investigations in Iran that have assessed the impact of occupational exposures on cancer outcomes. Although established epidemiological methods were used, their application to Iranian occupational exposure data has been limited. Our findings suggest that even with incomplete or heterogeneous national data, it is possible to generate exploratory estimates of the cancer burden attributable to major occupational carcinogens. Given the limited availability of reliable exposure and risk data, these estimates should be interpreted cautiously and considered preliminary.

The estimated number of cancer cases attributable to the examined occupational exposures was 1,285. Under a hypothetical scenario assuming 50% underreporting of occupational exposure, this number increased to 2,571 cases. Bladder, lung, leukemia, laryngeal, and liver cancers ranked first to fifth, respectively, in terms of attributable cancer cases related to occupational exposures. This pattern is broadly consistent with the global distribution of cancers commonly associated with occupational risk factors [45]. The highest attributable fractions were observed for asbestos (42%), silica (36%), lead (34%), and benzene (23%). The total number of the five cancers attributed to silica exposure was 623 cases, with bladder cancer being the most frequent (204 cases) and liver cancer the least (58 cases). These findings are influenced by assumptions regarding exposure prevalence and reporting completeness and should therefore be interpreted with caution.

Several international studies provide context for our findings. In the United Kingdom, Lesley Rushton et al.(2012) reported 907 lung cancer cases attributable to silica exposure during 2004–2005 [7]. In France, Micallef et al. (2018) estimated 444 lung cancer cases [23], while Houot et al. (2021), using a methodology similar to ours, reported 505 cases (95% UI: 254–753) [45], which is nearly equal to the total number of all cancers attributed to silica exposure estimated in our study. Our estimate for all cancers attributable to silica exposure in Iran is lower; however, direct comparisons should be interpreted cautiously because methodological differences, underreporting, and variations in exposure assessment may substantially influence the estimates. In developing countries, limited protective measures and incomplete exposure surveillance may also contribute to underestimation of the true burden [15, 46] .

Asbestos exposure in Iran showed a relatively high AF (42%), but the estimated number of attributable cases was very small (approximately 0.4 cases) because only a limited number of workers (*N* = 57) were reported to be exposed. This reflects the combined effect of a small exposed population and the assumptions used in the simulation model. International comparisons illustrate the broader context of asbestos-related cancer burden. In France, Marant Micallef et al.(2023) estimated 42 laryngeal cancer cases and 1,373 lung cancer cases attributable to occupational asbestos exposure [16], whereas Rushton et al. (2012) reported 2,223 lung cancer cases and 8 laryngeal cancer cases in the United Kingdom [7]. Similarly, Alain Lacourt et al. identified asbestos as the leading occupational contributor to lung cancer burden in France [47].

Although benzene exposure affects a relatively large worker population, the corresponding odds ratios were comparatively modest, resulting in a relatively high attributable number but lower AF compared with asbestos and silica exposures. Previous Iranian studies have reported lower attributable fractions for leukemia related to occupational benzene exposure [22], likely reflecting differences in methodology and exposure assessment approaches.

International comparisons show a similar pattern: Micallef et al. (2023) in France reported 41 leukemia cases attributable to occupational exposure [16], while Rushton et al.(2012) reported 7 cases in the United Kingdom [7], and Houot et al. (2021) estimated 16 cases in France [45]. In addition, Paolo Boffetta et al. (2010) reported 145 leukemia cases attributable to occupational exposure in France [6].Although our estimates were somewhat higher than those reported in several European studies, this may plausibly reflect the extensive oil, gas, and petrochemical industries in Iran, where occupational benzene exposure remains relatively common. Nevertheless, cross-country comparisons should be interpreted cautiously because differences in exposure prevalence, surveillance systems, modeling assumptions, and selected effect estimates can substantially influence attributable burden estimates.

This study appears to be the first national effort to estimate the burden of occupational cancers attributable to silica, asbestos, lead, and benzene in Iran using a unified analytical framework. A triangulation approach was used to integrate evidence from multiple sources, which is particularly useful in settings with limited high-quality exposure data. This approach allowed us to generate preliminary estimates while simultaneously identifying major evidence gaps.

The sensitivity analysis demonstrated the importance of exposure ascertainment in burden estimation. When reporting sensitivity was reduced from 100% to 50%, the estimated number of attributable cancer cases approximately doubled. This proportional increase suggests that the estimated burden is highly sensitive to assumptions regarding reporting completeness and highlights the importance of strengthening occupational exposure surveillance systems in Iran.

Several limitations should be considered when interpreting these findings. First, only four occupational carcinogens were systematically recorded in the national surveillance system, while several other established or probable carcinogens were not included. Second, exposure levels were not directly measured, and substantial uncertainty likely exists regarding the true prevalence and intensity of occupational exposures. Third, the modeling framework relied on several assumptions, including absence of confounding and interaction effects, fixed population structure, linear exposure–response relationships, and deterministic relationships among variables. In addition, effect estimates were derived largely from international studies conducted in developed countries, which may not fully reflect occupational conditions in Iran.

Finally, the uncertainty intervals presented in this study reflect variability propagated through the model inputs rather than full epidemiological uncertainty. Therefore, the results should not be interpreted as precise burden estimates. Instead, they represent exploratory, scenario-based estimates intended to provide an initial approximation of the potential occupational cancer burden in Iran and to support future surveillance, exposure assessment, and epidemiological research efforts.

## Conclusion

In conclusion, this study provides scenario-based estimates of the potential burden of occupational cancer in Iran and highlights the possible contribution of major occupational carcinogens to selected cancer outcomes. The findings suggest that historical workplace exposures to carcinogenic agents may contribute meaningfully to the national cancer burden and underscore the importance of preventive strategies in occupational settings where such exposures persist. Given the exploratory nature of the modeling approach and the limited availability of exposure and surveillance data, the estimates should be interpreted with caution and should not be considered precise estimates of burden. Nevertheless, this study provides an initial framework for understanding the occupational cancer burden in Iran and may help inform future surveillance, research, and occupational health policy.

## Supplementary Information

Supplementary Material 1.

Supplementary Material 2.

## Data Availability

The datasets generated during and/or analyzed during the present study can be inquired from the corresponding author on reasonable request.

## Abbreviations

AF: Attributable Fraction
OR: Odds Ratio
RR: Relative Risk
AN: Attributable Number
CI: Confidence Interval
UI: Uncertainty Interval
IARC: International Agency for Research on Cancer
BTEXs: Benzene, Toluene, Ethylbenzene, Xylenes
