# Supplementary material for: Attributable fraction and number of cancer cases attributed to occupational carcinogens in Iran in 2019
Source: BMC Public Health. 2026 Jun 25;26:2676. doi: 10.1186/s12889-026-28144-z (PMC13602639; doi:10.1186/s12889-026-28144-z)
Supplement: Supplementary file 1 [file 12889_2026_28144_MOESM1_ESM.docx]

**Supplementary Table S1.**Pooled odds ratios used exclusively for meta-regression assessment of heterogeneity across cancer types for each occupational exposure

| **OR (95%CI)** | **Included studies (N)** | **Agent** |
| --- | --- | --- |
| 1.34 (1.20–1.50) | 5 | Silica dust, crystalline |
| 1.39 (1.20–2.6) | 6 | Asbestos |
| 1.24(0.95–1.62) | 3 | Lead |
| 1.16 (1.04–1.29) | 3 | Benzene |
