# Supplementary material for: Attributable fraction and number of cancer cases attributed to occupational carcinogens in Iran in 2019
Source: BMC Public Health. 2026 Jun 25;26:2676. doi: 10.1186/s12889-026-28144-z (PMC13602639; doi:10.1186/s12889-026-28144-z)
Supplement: Supplementary file 2 [file 12889_2026_28144_MOESM2_ESM.docx]

**Dear interviewee,**

Thanking you for your cooperation, this interview is being conducted to identify and estimate the percentage of small, large, and medium-sized industries exposed to risk levels one, two, and three (based on the Ministry of Health's risk grading in terms of the type of harmful and carcinogenic exposures) for use in the doctoral thesis in epidemiology titled "Investigating the direct effect of occupational exposure in industrial environments on the incidence of cancer in Iran." It is hoped that desirable results can be achieved from the outcome of this interview.

Part 1: General Questions:

1- Interviewee's name

2- Educational qualification

3- Organizational position title

4- Place of employment

5- Number of years of employment

**Part 2:** The most common carcinogenic exposures in industries

Please express your opinion on the type and number of the most common carcinogenic exposures in Iranian industries.

**Part 3:** Specific questions on risk rating and type of exposure based on targeted inspection and risk rating by the Ministry of Health (level 1, level 2 and level 3):

|  | **Small size(≤25 worker)** | **Medium size (>25 to ≤250 worker)** | **Large size(≤250)** |
| --- | --- | --- | --- |
| **Number of industries covered** |  |  |  |
| **Number of employed workers** |  |  |  |
| **Percentage and number of workers at risk level 1** |  |  |  |
| **Percentage and number of employees at risk level 2** |  |  |  |
| **Percentage and number of employees at risk level 3** |  |  |  |
| **Percentage and number of workers controlling level 1 harmful factors** |  |  |  |
| **Percentage and number of workers controlling level 2 harmful factors** |  |  |  |
| **Percentage and number of workers controlling level 3 harmful factors** |  |  |  |
